# Guidelines for Genome-Wide Association Studies

**DOI:** 10.1371/journal.pgen.1002812

**Published:** 2012-07-05

**Authors:** Gregory S. Barsh, Gregory P. Copenhaver, Greg Gibson, Scott M. Williams

**Affiliations:** 1 HudsonAlpha Institute for Biotechnology, Huntsville, Alabama, United States of America; 2 Department of Genetics, Stanford University School of Medicine, Stanford, California, United States of America; 3 Department of Biology, The University of North Carolina at Chapel Hill, Chapel Hill, North Carolina, United States of America; 4 Carolina Center for Genome Sciences, The University of North Carolina at Chapel Hill, Chapel Hill, North Carolina, United States of America; 5 Center for Integrative Genomics, School of Biology, Georgia Institute of Technology, Atlanta, Georgia, United States of America; 6 Center for Human Genetics Research, Vanderbilt University, Nashville, Tennessee, United States of America

Genome-wide association studies (GWAS) have revolutionized human genetics. They have led to the identification of thousands of loci that affect both normal variation and susceptibility to disease, and have clarified our understanding of the genetic architecture of complex traits. In just five years, the methodology has moved from extraordinary to commonplace, and with the advent of affordable genome-scale data collection, GWAS and other genomic technologies are being adopted by model organism researchers. As GWAS applications have evolved so too have the community standards that pertain to them. Here we clarify the editorial policy of *PLoS Genetics* with regard to these guidelines.

Our overall goal—that is, the goal of our editorial board—is to emphasize work in which genetic approaches and genetic logic help us learn more about biology. As such, we are committed to publishing work in which the conclusions are both broadly significant and analytically rigorous. For studies in which a GWA approach is central, “broadly significant” means that the basic science, biomedical, agricultural, and/or social impact will be of substantial and interdisciplinary interest. For example, in well-studied diseases, the identification of a small number of additional loci whether in a novel sample or by meta-analysis will usually be considered more appropriate for a specialized audience. Regardless of the trait, we will be most enthusiastic about work that includes experiments or genetic analyses that address the mechanism by which a GWAS-based variant gives rise to phenotypic differences.

By “analytically rigorous”, we mean that controlling for multiple comparisons, population stratification, relatedness, and technical quality is critical. For work focused on gene discovery, minimizing the false positive rate is a more important consideration than controlling the false negative rate. In most of these cases, genome-wide significance thresholds (nominal *p*-value < 5×10̂-8 for a conventional GWAS) will be required, ideally accompanied by independent replication and analyses that include consideration of the joint as well as the individual discovery and replication datasets. For work focused on genetic architecture (for example, to understand the extent to which epistatic and/or gene–environment interactions control phenotypic variation), controlling for both type I and type II error is important, with the overall goal of ensuring that a robust statistical approach leads to an advance that is broadly significant.

What about model organisms or non-human natural populations? Here, the experimental systems are diverse, and include structured populations of plants and animals from around the world, large F2 or advanced intercrosses, recombinant inbred collections, and many non-model organisms for which there are important and interesting traits that provide biological insight. Overall, when genome-wide information is used to analyze these studies, we will require genome-wide significance thresholds analogous to those used in human studies in order to minimize the chance of false positive results. However, each system may be different, and will require careful evaluation to avoid or control for confounding, together with convincing evidence that the statistical model being employed fits the data being evaluated. In many situations, it will be appropriate to independently replicate genotype–phenotype correlations in the same way that we expect for human genetic studies.

Regardless of the species or the population being studied, genetic arguments should stand on their own. In many experimental organisms, proving a causal role for a particular variant observed in an association study can be accomplished by transgenic approaches, a form of independent replication. In other situations, including most natural populations, the situation is more complicated, and the extent to which whole animal phenotypes can be recapitulated in functional studies carried out in cells or tractable model organisms will require careful assessment and judgment by editors and reviewers. Importantly, we consider additional studies (such as bioinformatics, network analyses, and/or biochemical or cell biologic experiments) as work that can add to the strength of advance but not substitute for rigor of the quantitative genetic conclusions.

Finally, a central tenet of the Public Library of Science (PLoS) is open access, not only to the work being described, but also to the data being analyzed. Unless there are compelling—usually ethical—reasons to the contrary, complete datasets of both genotype and phenotype should be immediately available without restrictions. For human research, some restrictions on data availability may be necessary in order to respect privacy, or to prevent identification of participants; in these situations, and in meta-analyses, we expect policies to evolve in such a way that the benefit to the scientific community of data availability is balanced with the need to maintain high ethical standards.

We intend for these considerations to serve as guidelines rather than dogmatic requirements; indeed, hallmarks of *PLoS Genetics* are the autonomy of judgment and wisdom of consensus that stem from our editorial board structure. But just as the community benefits from consensus it will also benefit from consistency and rigor, and we hope that these guidelines will further the positive impact that GWAS can have on our understanding of the natural world and human health.

